# Beyond the beauty of occlusion: medical masks increase facial attractiveness more than other face coverings

**DOI:** 10.1186/s41235-021-00351-9

**Published:** 2022-01-10

**Authors:** Oliver Hies, Michael B. Lewis

**Affiliations:** School of Psychology, Cardiff University, Cardiff, UK

**Keywords:** Facial attractiveness, Medical face masks, Face occlusion effects

## Abstract

The sanitary-mask effect (Miyazaki and Kawahara in Jpn Psychol Res 58(3):261–272, 2016) is the finding that medical face masks prompt an image of disease and thus result in lower ratings of facial attractiveness of the wearer. However, during the COVID-19 pandemic, medical masks have been found to increase attractiveness (Patel et al. in Plast Reconstruct Surg Glob Open 8(8), 2020) although this could have been a general effect of occlusion. To further explore this issue, female participants were presented with a series of male faces of low or high attractiveness that were occluded with a medical mask, cloth mask, book or not occluded and asked to rate them on attractiveness. The results show that faces were considered as most attractive when covered by medical masks and significantly more attractive when occluded with cloth masks than when not occluded. Contrary to expectation, base attractiveness did not interact with the type of occlusion, suggesting that this is not simply due to occlusion of negative features. The present findings are contrary to the sanitary-mask effect and explanations in terms of social desirability, and the association of medical masks with caregiving professions is explored.

## Introduction

As a result of the outbreak of the Coronavirus disease 2019 (COVID-19) pandemic, there has been a sharp increase in demand for protective face masks globally to prevent the spread of the virus. Since July 2020 (Rab et al., 2020), it was mandatory to wear masks covering the mouth and nose in the UK in a number of different places, for example supermarkets and on public transport—although, rules changed after June 2021. Despite concerns about masks eliciting a false-sense of security (Javid et al., 2020), research shows they are of paramount importance in helping to reduce the spread of COVID-19 (Howard et al., 2021), which is reflected in government policy worldwide.

Notwithstanding the medical benefits, the use of masks carries costs. Evidence suggests wearing masks induces perceived discomfort, impairs vocal communication (Ribeiro et al., 2020), and may be a barrier to social interaction (Hung, 2018). Furthermore, masks prevent crucial face-to-face interactions between newborns and their primary attachment figures, which could impede early bonding (Green et al., 2020). Finally, research shows masks impair recognition of emotions (Carbon, 2020), with participants reporting lower confidence and greater patterns of confusion. Overall, research on side effects of masks has significant implications for day-to-day life, in which recognising emotions and clear communication are key to interpersonal interaction.

While face coverings were a somewhat rare sight in Western nations, such as the UK, prior to 2020, they have been common in some cultures long before the COVID-19 pandemic. Many Muslim women wear niqābs or burqas, which cover large parts of the face. Medical masks (sometimes referred to as surgical or sanitary masks) are routinely used for health reasons in collectivist countries such as Japan, where people have enhanced personal hygiene practices (Wada et al., 2012). Masks are also customary in China as a protection from air pollution, despite concerns about their efficacy (Cherrie et al., 2018).

## Face masks and attractiveness

In contrast to the problems associated with masks discussed above, there is a common belief among Japanese women that wearing a mask increases one’s attractiveness, as it occludes potentially undesirable facial features such as acne (Miyazaki & Kawahara, 2016). Wearing masks in Japan was common long before the COVID-19 pandemic, as many believe it reduces transmission of influenza viruses (Burgess & Horii, 2012). Miyazaki and Kawahara (2016) thus investigated the effect of wearing a medical mask on perceived facial attractiveness in a Japanese sample. They conducted a series of experiments in which they presented participants with images of female faces previously rated as high, low or medium in attractiveness. Some faces were wearing medical masks, while others had the lower half occluded by a card or notebook (control occluders) and further faces were not occluded. Participants’ task was to rate the faces on attractiveness. The authors found that faces occluded by a medical mask were perceived as less attractive than those that were not (regardless of base attractiveness), which they termed the *sanitary-mask effect* (Miyazaki & Kawahara, 2016, p. 262). In contrast, this effect was not found for faces covered by control occluders. Instead, the authors found an occlusion effect, which is a regression to the mean, with attractiveness ratings increasing for faces low in base attractiveness and decreasing for faces high in base attractiveness. The latter finding is plausible, as covering the lower half of the face is likely to obstruct undesirable or desirable facial features (Miyazaki & Kawahara, 2016); however, occlusion of facial features has been found to increase facial attractiveness regardless of which features are occluded and the base attractiveness of the face (Sadr & Krowicki, 2019). Miyazaki and Kawahara (2016) explain the sanitary-mask effect in terms of medical masks acting as a prime of disease, which is supported by data obtained in a survey, which showed that people associated medical masks with disease. Moreover, the authors found that masked faces were consistently perceived as less healthy than unmasked faces. Overall, these findings suggest medical masks are linked to poor health, and individuals wearing them are seen as less attractive. This is consistent with the idea of a behavioural immune system where disease-related stimuli are automatically associated with unattractiveness (e.g. Klebl et al., 2021).

With the outbreak of the COVID-19 pandemic, more research has been conducted in this area. Patel et al. (2020) investigated the influence of facial occlusion with medical masks on judgments of facial attractiveness in the United States. Without reference to Miyazaki and Kawahara’s (2016) research, and contrary to what the sanitary-mask effect would predict, the authors hypothesised that faces wearing medical masks would be rated as more attractive than unmasked faces due to the importance of lower face information, particularly from the perioral area, in determining facial attractiveness. Patel et al. (2020) found that unattractive and averagely attractive faces were rated as significantly more attractive when in the masked compared to unmasked condition. This effect was greatest for faces in the unattractive condition across both males and females. Attractive faces were rated as less attractive in the masked condition. These findings reflect the occlusion effect found by Miyazaki and Kawahara (2016) in their control occluder condition. Patel et al. (2020) did not include an occlusion control condition and so it is not possible to distinguish between the effect of occlusion and the effect of the medical mask in their study and so it is possible that the sanitary-mask effect is still present but swamped by the occlusion effect.

In related research, Olivera-La Rosa et al. (2020) looked at the effect of multiple individual differences on, among other variables, illness and social desirability perceptions of faces with or without medical masks. This took place in Spanish-speaking countries in Europe and South America. They found that people shown as masked faces were generally considered more socially desirable than unmasked faces. Furthermore, they found that masked faces were also rated as more likely to be ill than unmasked faces, which supports Miyazaki and Kawahara’s (2016) findings. As previous research has shown, social desirability and physical attractiveness are closely linked (Huston, 1973); these findings raise the question as to whether facial attractiveness and health can be dissociated. One possible explanation for these counterintuitive results is that participants formed a more implicit association between medical masks and illness, but a deliberative link between medical masks and social desirability, which may reflect the role of social norms in attractiveness judgments (Olivera-La Rosa et al., 2020). A link between medical-related attire and attractiveness has previously been shown by Braise and Richmond (2004) who found that male doctors in white coats were judged as more attractive than those not in white coats. The medical face mask has, in fact, become an icon of the medical professionals’ fight against the COVID-19 pandemic as illustrated by the artwork of Nathan Wyburn (Wyburn, 2020). Thus, the social desirability of medical mask wearers could potentially off-set the association with disease in the assessment of the attractiveness of faces with medical masks on.

Finally, the original Miyazaki and Kawahara (2016) experiment was repeated during the pandemic in Japan (Kamatani et al., 2021). In this study, the sanitary-mask effect disappeared and there was only a regression to the mean such that the mask made unattractive faces more attractive and attractive faces less attractive. This study used either black or white masks rather than the more typical blue medical masks. Also, and as in Patel et al. (2020), there was no occlusion-only control condition. There was a decrease in the association of mask wearing and perceived unhealthiness between the pre-pandemic and pandemic versions of the experiments, indicating that COVID-19 could be underpinning this shift in the perception of attractiveness of faces with masks.

## The present study

The present study set out to answer a number of questions related to facial attractiveness and occlusion that have arisen from previous research, in particular from Miyazaki and Kawahara’s (2016) findings. Firstly, how universal and robust is the sanitary-mask effect? Does it generalise to the UK population during a global pandemic? Secondly, how do cloth face masks affect judgments of facial attractiveness? Reusable cloth masks are a much more common sight in the UK at the moment and so does this increased familiarity have an impact on assessment of the wearer? Finally, acceptance of mask wearing is not universal and it was explored whether attitudes towards mask wearing impact peoples’ assessment of the mask-wearer. To investigate these questions, the present study employed a 2 × 4 within-subjects design with the independent variables (IVs) base attractiveness and type of occlusion and the dependent variable (DV) facial attractiveness. Participants were asked to rate the attractiveness of faces previously rated as unattractive or attractive (low vs high base attractiveness) when occluded with a medical mask, occluded with a cloth face mask, occluded with a control object, or not occluded.

## Method

### Participants

An a priori power analysis used an appropriate effect size obtained from Miyazaki and Kawahara (2016). Given an eta-squared score of 0.22 (medical mask effect) and an alpha level of 0.05, a within-participant study with just 10 participants would give a power of 0.8. Given that there were more comparisons being made here than the simple mask/no-mask comparison, the number of participants was increased to 43. Thus, 43 Cardiff University undergraduate Psychology students were recruited on the School of Psychology’s Experimental Management System. The study was only available to female participants. One participant was excluded due to arbitrary responding. In the final pool of participants, most (40) indicated their age as being between 18 and 24 years. The majority of participants (40) identified as white.

### Materials

The stimuli were generated from images of 40 different male faces (2444 × 1718 pixels) from the Chicago Face Database (CFD) Version 2.0.3—July 2016 (Ma et al., 2015). All images were frontal portraits. The faces varied in race and physical attractiveness. The 20 most attractive and 20 least attractive faces, based on previous ratings included in the CFD, were selected. The mean previous rating for the high attractiveness faces was 4.26 (SD = 0.32) on a seven-point Likert scale (1 = Not at all, 7 = Extremely), and the mean for the low attractiveness faces was 2.06 (SD = 0.19) (Ma et al., 2015). The perceived age of the faces was between 18 and 30 years (*M* = 24.94, SD = 2.96). Only neutral facial expressions were chosen. While the faces differed in hairstyle, none had piercings, glasses, beards, or other potentially occluding features or confounding factors.

Occluding stimuli were made from frontal portraits of a model wearing three different cloth face masks and a medical mask. The model was also photographed holding a plain, black book covering a comparable area of the face as masks do. The cloth masks were made of cotton and characterised by abstract patterns.

**Fig. 1:**
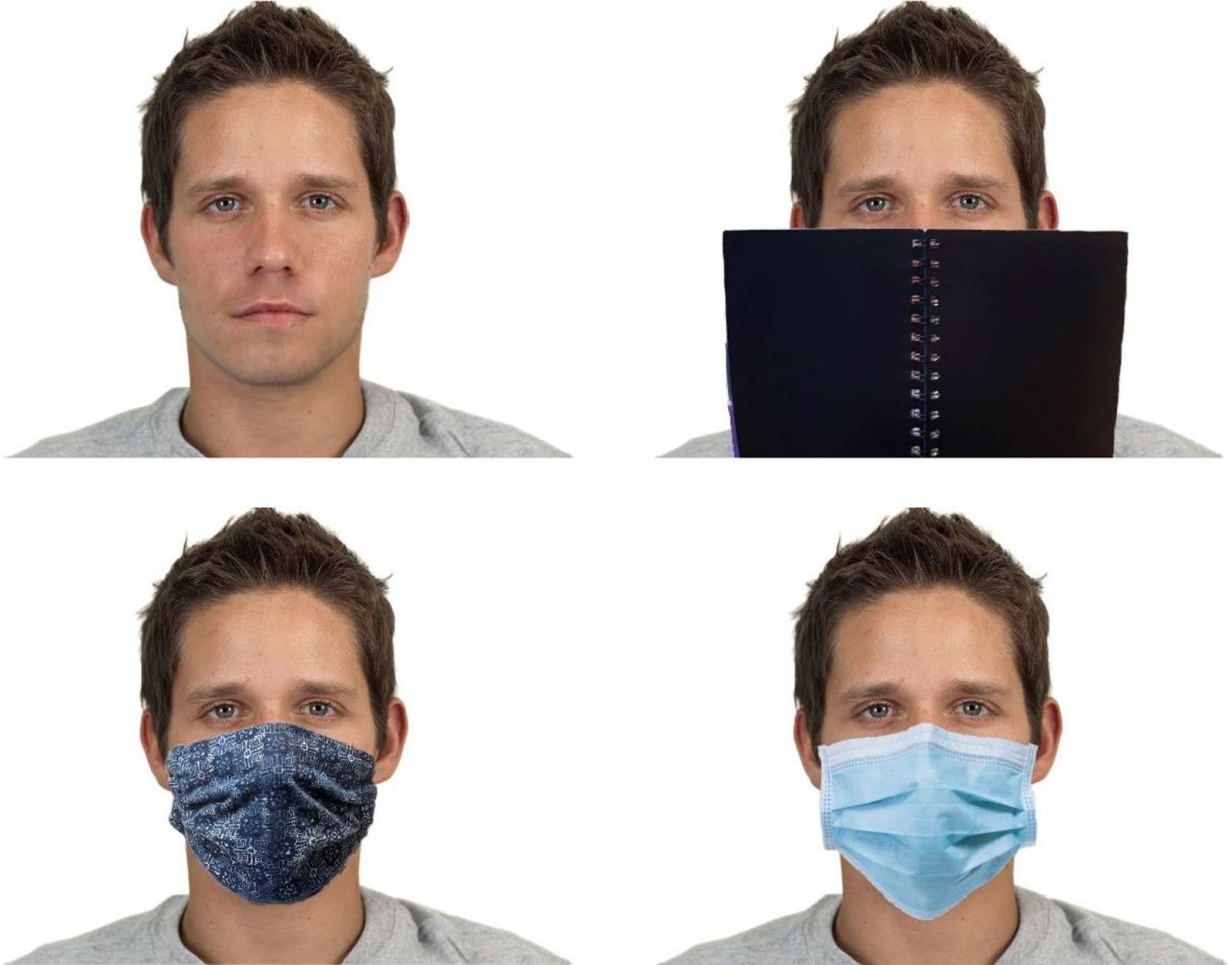
Examples of the four types of stimuli used. Top left: Full face. Top right: Notebook. Bottom left: Cloth mask. Bottom right: Medical mask. The original CFD image is reproduced with permission

The final stimuli were constructed from the 40 CFD faces with each of the three types of occluding stimuli. (Only one cloth mask was used for each face.) The facial occluders were adjusted using editing software to make sure the digitally created stimuli appeared as natural and consistent as possible and covered the same facial proportions across different faces. The edges of the occluders were edited to make sure they blended in well with the rest of the face. Thus, there were 160 different stimuli, i.e. 40 different faces with no facial occluder, a medical mask, a cloth mask and a book occluder, as depicted in Fig. 1.

### Procedure

The experiment was set up on PsychoPy and run in February 2021, i.e. seven months after wearing masks became mandatory in the UK. Due to COVID-19 restrictions, it was conducted online via Pavlovia.org and thus completed on participants’ own devices. Participants were asked to complete the experiment in a quiet and suitable environment to minimise sources of distraction.

Participants were told they would be presented with a number of male faces and instructed to rate them on facial attractiveness on a scale from 1 to 7. To familiarise participants with the experiment, an example trial with a face from the CFD that was not used in the main experiment was included. The 160 stimuli described above were then individually presented to participants in a random order. Therefore, each participant was presented with each face four times (medical mask, cloth mask, book, non-occluded). Participants rated the faces using the number keys 1–7.

After rating the faces, participants were asked to indicate their age, ethnicity and level of agreement on a five-point Likert scale to the following two statements assessing attitudes towards face masks: “Face masks have become part of everyday life in the past year” and “The use of face masks is effective in preventing the spread of COVID-19”. Finally, participants were debriefed and received course credit as compensation for their participation.

### Design

This study used a 2 × 4 within-subjects design. The independent variables were type of occlusion (four levels: medical mask, cloth mask, notebook, and no occlusion) and base attractiveness (low vs high). The dependent variable was participants’ facial attractiveness ratings, which were measured using a seven-point Likert scale ranging from *very unattractive* to *very attractive*.

## Results

**Fig. 2:**
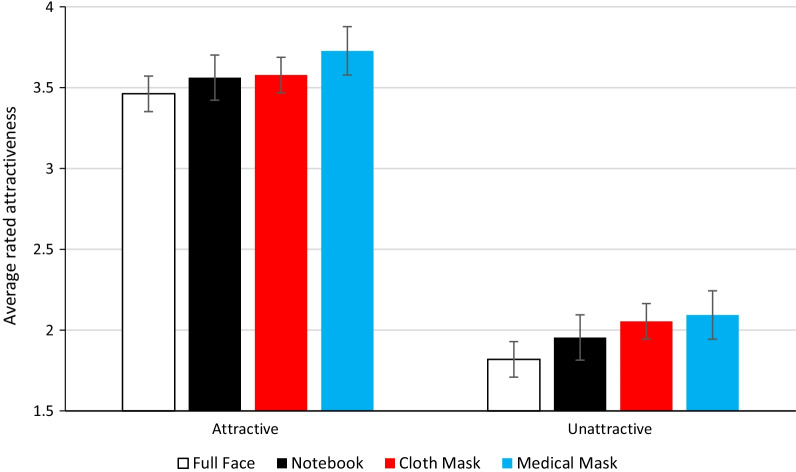
Mean attractiveness ratings for the four occlusion conditions for the faces that were initially rated as attractive or unattractive. The error bars show ± one standard error calculated from within participant variance

The full dataset is available at https://osf.io/9p5q7/. The mean attractiveness ratings for each of the four occlusion conditions are presented below (Fig. 2). They show that faces low in base attractiveness were rated as less attractive than those high in base attractiveness across types of occlusions, but there are also smaller effects of occlusion type.

The data were analysed using a two‐way repeated-measures analysis of variance (ANOVA). As sphericity was violated for the effect of occlusion, a Greenhouse–Geisser adjustment was applied. The analysis revealed a main effect of base attractiveness, with faces high in base attractiveness rated as significantly more attractive than those low in base attractiveness: *F*(1, 40) = 235.94, *p* < .001, *η*_*p*_^2^ = .86. Moreover, a main effect of occlusion was found: *F*(2.35, 94.29) = 19.04, *p* < .001, *η*_*p*_^2^ = .32 (see Fig. 2). The analysis did not reveal a statistically significant interaction effect between base attractiveness and occlusion: *F*(2.82, 113.07) = 1.52, *p* = .198, *η*^2^ = .04).

Pairwise comparisons (Holm–Bonferroni corrected) of the different occlusion conditions show that faces were rated as significantly more attractive in the medical mask condition compared to in the cloth mask condition (*p* = .020), notebook occluder condition (*p* < .001), and control condition (*p* < .001). In addition, faces in the cloth mask condition were rated as significantly more attractive than in the control condition (*p* < .001), but they were only non-significantly more attractive than the notebook condition (*p* = .123). Further, faces in the notebook condition were rated as significantly more attractive than in the control condition (*p* = .005).

Finally, participants showed high levels of agreement with the statements “Face masks have become part of everyday life in the past year” (*M* = 4.62, SD = 0.94) and “The use of face masks is effective in preventing the spread of COVID-19” (*M* = 4.26, SD = 0.89). This meant that investigating correlations of attractiveness differences with mask-wearing attitudes was not possible.

## Discussion

The results run counter to Miyazaki and Kawahara’s (2016) sanitary-mask effect: faces occluded with a mask were seen as more attractive compared to those that were non-occluded. Surprisingly, there was no evidence of a regression to the mean through occlusion as indicated by the lack of a significant interaction. The size of the mask or occlusion effects was roughly equivalent for attractive and unattractive faces. The advantage for occluded faces is consistent with the general finding that occlusion improves attractiveness (Sadr & Krowicki, 2019). Furthermore, this finding is consistent with Patel et al.’s (2020) findings, which also emphasise that medical masks increase facial attractiveness for unattractive and average faces.

While the present findings suggest that cloth masks enhance facial attractiveness, there appears to be an advantage to medical masks beyond this. The advantage for a cloth mask can be attributed to the effect of occlusion, but the effect of the medical mask goes beyond just hiding undesirable features. It is possible that the additional advantage for medical masks comes from their associations with medical professionals. It has been shown, for example, that females rate male doctors as more attractive if they wear a white coat (Brase & Richmond, 2004). A similar association with the person being in a caring profession could explain the advantage for faces wearing medical masks. This white-coat effect could also potentially explain the contrast between Miyazaki and Kawahara’s (2016) findings and the ones reported here. Brase and Richmond (2004) found that the white-coat effect on attractiveness was reversed for female faces, and Miyazaki and Kawahara (2016) used mostly female faces, whereas only male faces were employed here. While medical masks might prime disease, they can also be seen as a sign of being responsible and caring citizens, which may positively impact perceived attractiveness. This notion is in line with Olivera-La Rosa et al.’s (2020) research, which shows faces wearing medical masks are considered more likely to be ill, but also more socially desirable and trustworthy. The fact that the participants here reported favourable views on the effectiveness of masks in preventing the spread of COVID-19 adds weight to this possible explanation. It is possible that Miyazaki and Kawahara’s (2016) participants did not see the social responsibility element of mask wearing and so the association with disease had a larger effect than the positive effects of medical mask wearing.

From the results presented here, and previous results, there appear to be at least three effects at play in the interaction between face masks and attractiveness. The sanitary-mask effect is the reduction of facial attraction produced by the association between the mask and disease. This effect could be restricted to Japanese faces, and the COVID-19 pandemic has reduced its influence. The occlusion effect is the increase in attractiveness produced simply by obscuring the lower part of the face. This effect can be produced by any object and is not dependent on a mask. A further medical-mask effect is observed here whereby medical masks increase facial attractiveness, maybe because of the association of the masks with the medical or caring professions. This effect may only be present during the COVID-19 pandemic and, so far, has only been shown for male faces. Combinations of these three different effects are able to explain the disparate findings presented here and also previous research in the field. The exact relative contribution of these three effects across genders, cultures and global health crisis remains to be fully explored.

## Data Availability

The datasets generated and/or analysed during the current study are available via the Centre Open Science repository, https://osf.io/fgp3j/.

## References

[CR20] Brase GL, Richmond J (2004). The white–coat effect: Physician attire and perceived authority, friendliness, and attractiveness. Journal of Applied Social Psychology.

[CR1] Burgess A, Horii M (2012). Risk, ritual and health responsibilisation: Japan’s ‘safety blanket’ of surgical face mask-wearing. Sociology of Health & Illness.

[CR2] Carbon CC (2020). Wearing face masks strongly confuses counterparts in reading emotions. Frontiers in Psychology.

[CR3] Cherrie JW, Apsley A, Cowie H, Steinle S, Mueller W, Lin C, Loh M (2018). Effectiveness of face masks used to protect Beijing residents against particulate air pollution. Occupational and Environmental Medicine.

[CR4] Green J, Petty J, Staff L, Bromley P, Jones L (2020). The implications of face masks for babies and families during the COVID-19 pandemic: A discussion paper. Journal of Neonatal Nursing.

[CR5] Howard, J., Huang, A., Li, Z., Tufekci, Z., Zdimal, V., van der Westhuizen, H. M., & Rimoin, A. W. (2021). An evidence review of face masks against COVID-19. *Proceedings of the National Academy of Sciences*, *118*(4), e2014564118. 10.1073/pnas.2014564118. 10.1073/pnas.2014564118 PMC7848583 33431650

[CR6] Hung YW (2018). A study of barriers to the wearing of face masks by adults in the US to prevent the spread of influenza.

[CR7] Huston TL (1973). Ambiguity of acceptance, social desirability, and dating choice. Journal of Experimental Social Psychology.

[CR8] Javid, B., Weekes, M. P., & Matheson, N. J. (2020). Covid-19: should the public wear face masks? *369*, m1442. 10.1136/bmj.m1442. 10.1136/bmj.m1442 32273278

[CR9] Kamatani, M., Ito, M., Miyazaki, Y., & Kawahara, J. I. (2021). Effects of masks worn to protect against COVID-19 on the perception of facial attractiveness. *i-Perception*, *12*(3), 1–14. 10.1177/20416695211027920. 10.1177/20416695211027920 PMC8243111 34262683

[CR10] Klebl C, Greenaway KH, Rhee JJS, Bastian B (2021). Ugliness judgments alert us to cues of pathogen presence. Social Psychological and Personality Science.

[CR11] Ma DS, Correll J, Wittenbrink B (2015). The Chicago face database: A free stimulus set of faces and norming data. Behavior Research Methods.

[CR12] Miyazaki Y, Kawahara JI (2016). The sanitary-mask effect on perceived facial attractiveness. Japanese Psychological Research.

[CR13] Olivera-La Rosa A, Chuquichambi EG, Ingram GP (2020). Keep your (social) distance: Pathogen concerns and social perception in the time of COVID-19. Personality and Individual Differences.

[CR14] Patel V, Mazzaferro DM, Sarwer DB, Bartlett SP (2020). Beauty and the mask. Plastic and Reconstructive Surgery Global Open.

[CR15] Rab S, Javaid M, Haleem A, Vaishya R (2020). Face masks are new normal after COVID-19 pandemic. Diabetes & Metabolic Syndrome: Clinical Research & Reviews.

[CR16] Ribeiro VV, Dassie-Leite AP, Pereira EC, Santos ADN, Martins P, de Alencar Irineu R (2020). Effect of wearing a face mask on vocal self-perception during a pandemic. Journal of Voice.

[CR17] Sadr J, Krowicki L (2019). Face perception loves a challenge: Less information sparks more attraction. Vision Research.

[CR18] Wada K, Oka-Ezoe K, Smith DR (2012). Wearing face masks in public during the influenza season may reflect other positive hygiene practices in Japan. BMC Public Health.

[CR19] Wyburn, N. [@NathanWyburnArt] (2020, March 29). WAIT FOR IT... my tribute to our @NHSuk A few days ago I asked for NHS workers to send me photos of them. The response was overwhelming. Retrieved from: https://twitter.com/NathanWyburnArt/status/1244367015730720768

